# Effect of Indoors Artificial Lighting Conditions on Computer-Based Learning Performance

**DOI:** 10.3390/ijerph17072537

**Published:** 2020-04-08

**Authors:** Rui Zhang, Yalong Yang, Qiansheng Fang, Yufu Liu, Xulai Zhu, Mingyue Wang, Liangliang Su

**Affiliations:** 1 Anhui Province Key Laboratory of Intelligent Building and Building Energy Saving, Anhui Jianzhu University, Hefei 230022, China; rzhang0551@foxmail.com (R.Z.); qsfang@sohu.com (Q.F.); fu_ly0810@163.com (Y.L.); zhuxulai@126.com (X.Z.); wmingyue198@sina.com (M.W.); sulanqing_sd@163.com (L.S.); 2 School of Electronic and Information Engineering, Anhui Jianzhu University, Hefei 230601, China

**Keywords:** attention status, EEG measurement, lighting conditions, learning efficiency

## Abstract

Lighting condition is essential to human performance. With the widespread use of computer-based learning, the performance measurements become difficult, and the effects of artificial lighting conditions towards the new learning forms are not investigated extensively. The current study conducts a subject-within experiment with a 45-min-long online learning along with electroencephalogram (EEG)-based measurements, and a post-interview under five lighting setups respectively (300 lx, 3000 K; 300 lx, 4000 K; 300 lx, 6500 K; 500 lx, 4000 K; 1000 lx, 4000 K). Attention is chosen as the key factor to represent the learning performance. The results show that the attention of people aged in the 20s is not affected by the experimental lighting conditions. The results also demonstrate that people in high illumination at 1000 lx are more inclined to sustain attention despite the discomfort and dissatisfaction. Taking the EEG-based attention measurements and post-interview answers into consideration, lighting conditions at 300 lx, 4000 K are the recommended set points for university architectures among the investigated conditions, providing a practical basis when adjusting the lighting standard for its advantage in energy saving.

## 1. Introduction

Human-building interaction is always a popular topic in the field of building and the environment. Factors including acoustic, thermal, and illumination are most widely studied for the significant effects on human working and learning performance [1,2,3,4]. It is identified that the lighting environment affects vision, circadian rhythms, mood, cognition, attention, etc. by continuous studies. Standards of lighting design vary from countries and scenes. Most rooms with learning function, like university classrooms and working offices, set the illumination higher than the baseline at 300 lx. Generally, control strategy with higher illumination set points and color temperature changeability cost more energy consumption. The necessity of setting a higher illumination value and changing the color temperature requires more research [5,6]. 

Based on the experiments with different lighting settings, various conclusions are drawn. In Baron’s research, lighting conditions that generated a positive affect have an influence on subjects’ behavior and cognition [7]. However, no lighting effects were found for motivation or concentration, according to Mott’s field study [8]. Feelings of alertness and vitality, as well as objective performance and physiological arousal, might be improved by more intense light [9] while long-term memory can be improved by low illumination rather than high illumination [10]. Meanwhile, lower lighting levels under 500 lx is acceptable without compromising the user experience [11]. Young Adults are more likely to preserve a negative mood under low color temperatures while accomplishing cognitive tasks [12]. On the contrary, high correlated color temperature fluorescent lights seem to improve wellbeing and productivity based on Mills’s work [13]. When the light is blue-enriched, which means a relatively high color temperature, subjective alertness, performance, and evening fatigue would be significantly improved [14]. Smolders et al. draws an opposite conclusion that no significant activating effects and even subtle performance-undermining effects in the relatively high CCT condition [15]. These lighting settings formed a wild feature space, while some set points are not common in practical scenes. When studying the learning performance, the realistic lighting setting scale of university buildings should be supported by further study.

It can be seen that different experiment settings lead to different conclusions on subjective performance and mental status. However, no matter whether the experiment is field-based or artificial chamber-based, the performance assessments are all based on test scores. These assessments are not suitable for the work, which requires comprehension and imagination, for example, the learning task because the scores are difficult to be made. The effects of artificial lighting settings on learning performance need to be investigated extensively. In recent research [2,16,17,18], electroencephalogram (EEG) started to be adopted when examining people’s mental state and performance. It is presumed that mapping for the relative Highbeta wave in the temporal lobe can be a biological marker for assessing whether an environment is stressful or not [19]. Besides, thermal pleasure is positively correlated with the relative theta powers at Fz, Cz position [20]. A specific ratio of beta wave and theta wave can also analyze people’s attention ability [21]. The application of EEG gives inspirations when studying the learning performance.

Previous literature chose simple tasks to get the subject’s concentration for the sake of investigating mental status [22,23,24]. The Stroup task, N-back task, and vigilance tasks are often adopted to test subjects’ attention status, which is assessed by the accuracy per time unit. The higher the accuracy rate is, the higher the attention status level is. When inspecting the effect induced by different Illumination conditions on people’s attention status within a school class-long period, it is improper to use the tasks mentioned above since the workload is too heavy to burden for the subjects. With task time increasing, people’s fatigue would be the leading factor that affects attention. The effect caused by the illumination conditions would be neglected. Besides, this task cannot represent normal school class learning. Considering this, online learning is adopted as a task. Online learning is a popular approach for people to get self-education. With the development of online education, scenes of computer-based learning occur more frequently in schools and residences. It develops rapidly in recent years in China. Many universities, including foreign universities, release teaching videos free through online platforms, such as MOOC (Massive Open Online Course), Pan-Asian, NetEase open course, and so on. Many courses are the core curriculum in higher education. The procession of learning these courses can better reflect the variation tendency of students’ attention during school class –long period.

This study aims to investigate the effect of artificial lighting conditions on computer-based learning performance. Subject-within experiments based on EEG measurement and post interviews are conducted to evaluate the computer-based learning performance from both objective and subjective sides. Illumination and color temperature are two factors of lighting conditions discussed here. The set points are 3000 K, 4000 K, 6500 K with a fixed illumination at 300 lx as well as 300 lx, 500 lx, 1000 lx at a fixed color temperature at 4000 K, five combined conditions in total. The computer-based learning performance is assessed by attention status, which is the key indicator of learning performance. Interviews are conducted to inspect the subjective perception of the lighting conditions for support. 

## 2. Materials and Methods 

### 2.1. Experiment 

The experiment was conducted in the chamber located in Anhui Jianzhu University, PR China. The chamber is 8.6 m in length, 4.5 m in width, and 2.6 m in height. A High precision air conditioner HADC0191 is equipped to control the indoor air temperature, relative humidity, wind speed, and ventilation. According to the ASHRAE 55 standard, the air temperature was set at 24 ± 1 ℃ for the best thermal comfort. The relative humidity was set at 50%, and the wind speed was set at 0.1 m/s. Windows were screened with thick curtains to eliminate the effect of daylight. In the experiment, illumination and color temperature were the two characteristics of the lighting environment taken into consideration. Illumination, which is measured in “lx” refers to the flux of visible light received per unit area. Color temperature, measured in “K” represents the color component of a ray. According to the national standard for lighting design of buildings, the minimum illumination in the office is set at 300 lx, 500 lx, and 1000 lx are also recommended in other working scenes. For the sake of investigating the computer-based learning scene in university buildings, these three illumination levels were set. Three color temperature levels were set at 3000 K, 4500 K, and 6000 K, corresponding to warm light, white light and cold light, respectively. These settings also represent the normal color temperature settings of LED in Chinese markets. Thus, we got five lighting conditions in total, which is shown in Table 1. In addition, the actual value measured at 75 cm high desk is also presented. When conducting the experiments, other environmental indices were kept relatively unchanged.

**Figure 1:**
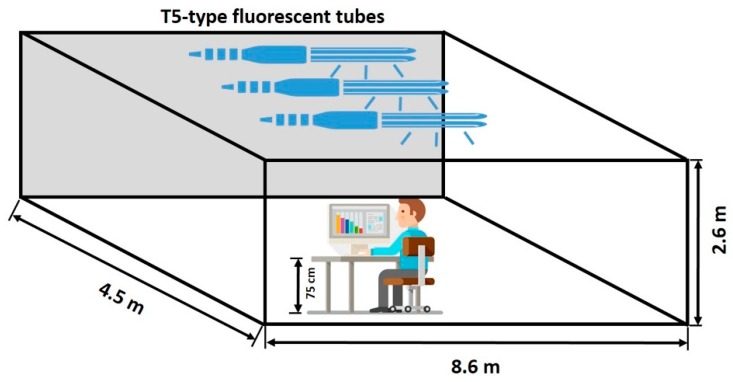
Fluorescent tubes setup.

To simulate the five different lighting conditions, T5-type fluorescent tubes were deployed, as is shown in Figure 1. The color temperature of the tubes was fixed with 3000 K, 4000 K, and 6500 K. The illumination was controlled by setting the number of working tubes. 

Eight male students from the university were recruited as human subjects. Female subjects were excluded from the experiment to eliminate the effect of the menstrual cycle. The average age was 20. All subjects had no history of serious diseases and abnormalities in Electrocardiograph (ECG), color blindness, alcohol abuse, smoking, and other bad habits. Before participating in the experiment, subjects were required to keep emotional stability, adequate sleep, and a good diet. The subjects were also asked not to smoke or drink alcohol, tea, or coffee that could stimulate nerves within 12 h before the test, and no vigorous exercise within 3 h. 

The experiment took around in September 2018 and May 2019, 9 am to 10 am and both phases were with the same procedure, which lasts for about 60 min. The subjects were asked to stay in the adjust room initially for 10 min to listen to a piece of gentle melody for the sake of restoring calmness and relaxation. Then they stepped into the chamber where the test illumination was already set, and the screen luminance was automatically adjusted according to the environment light. After all the preparation, a computer-learning task that needs the subjects to concentrate was assigned. Subjects were requested to watch a piece of video material for a common class period, and EEG was recorded during the video.

### 2.2. Task, EEG Data Collection, and Analysis as Well as Interview 

We chose the MOOC course to be the learning material. The contents of the courses were chosen according to the major of the subjects; usually the course arranged in the next semester to guarantee that the subjects are capable of concentrating on the courses with reasonable difficulty.

Electroencephalogram (EEG) is a general reflection of the electrophysiological activity of brain nerve cells on the surface of the cerebral cortex or scalp. It is adopted to record brain activity using electrophysiological markers, which sums up the postsynaptic potentials synchronously produced by a large number of neurons in the brain. Different mental activities would lead to variant EEG features, which offer the researchers an objective way to evaluate the subjects’ attention level. 

EEG composes of the five following frequency bands: Delta (0–4 Hz), Theta (4–7 Hz), Alpha (8–13 Hz), beta (13–30 Hz), gamma (30–50 Hz). Theta wave is associated with consciousness slips towards drowsiness. Beta wave appears when people are awake and having concentration. Through this frequency band, middle beta waves are specially recognized within the range of 16–20 Hz because of its strong correspondence to active mental activities. Besides, a frequency band called sensorimotor rhythm (SMR) waves that covers both alpha and beta waves within the range of 12–15 Hz is used when assessing the attention status. A ratio of the power of the three waves is adopted to analyze the subjects’ attention status, as shown in Equation (1) [18].

(SMR + Middle Beta)/Theta(1)

**Figure 2:**
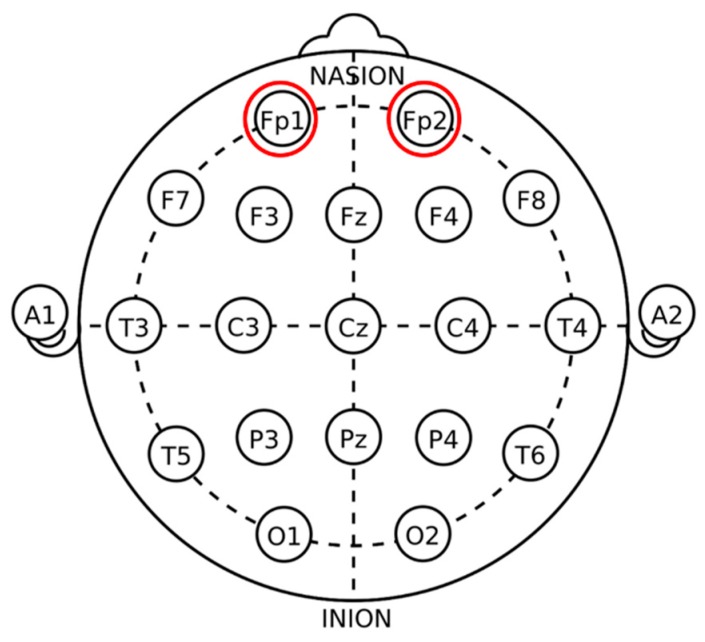
International 10–20 system of electrode placement.

In the experiment, EEG was sampled at 256 Hz by electroencephalograph (Brain Product) with the selected electrodes at the frontal region, FP1 and FP2, specifically shown in Figure 2. Signals were the average value of the two measured points. The 45-min task period was divided into nine segments with each for five minutes. Filtering and artificial removing were processed by the Brain vision analyzer, supporting analysis software, and spectral power was calculated by the EEGLab toolbox running under the Matlab environment.

Wilcoxon paired-signed ranks test was used for consistent comparison. Significance was accepted for values of *α* < 0.05. Several tests of EEG data under paired controlled lighting settings were made. The EEG data contained two types, EEG values in each sequence from 1 to 9 (45 min, 5 min per segment) and the average EEG values of the whole nine segments. Paired controlled lighting settings were between 3000 K, 300 lx and 4000 K, 300 lx; 4000 K, 300 lx and 6500 K, 300 lx; 4000 K, 300 lx and 4000 K, 500 lx; 4000 K, 500 lx and 4000 K, 1000 lx.

Interviews were conducted after the tasks. The extent of agreement towards four statements shown in Table 2 needs to be investigated. Scale from −2 to +2 is adopted. −2 refers to intense disagreeing and +2 refers to intense agreeing. In this section, the EEG collection was excluded.

The acquisition of subjective perception via this form rather than a questionnaire could eliminate the hasty assessment of each statement to a larger extent with the interaction between the subjects and the researchers. Direct and related questions were asked, and self-statements are encouraged to be given. During the assessment, the effect of lighting condition was emphasized to the subjects. For example, when discussing the first statement, whether their eyes were dry, whether they were feeling dizzy, and other body indications were explored. Besides, the subjects were asked to affirm whether the present lighting condition is appropriate if they were employed in entertainment now. This was also an important indicator when investigating comfort. When the subjects were uncertain about the scores, the researches would turn up and turned down the light on a small scale to check their tendency and made a more accurate score.

## 3. Results

### 3.1. Attention Analysis under Different Illumination Conditions

**Figure 3:**
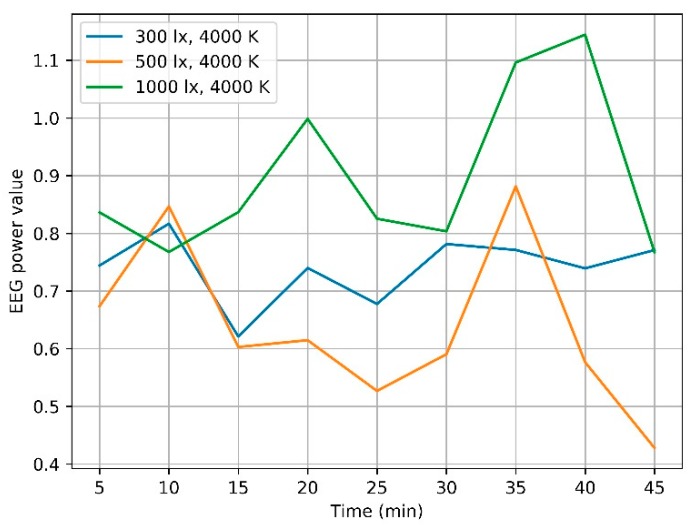
Attention status under different illumination conditions.

The variation of attention state calculated by Equation (1) is shown in Figure 3. Under the color temperature at 4000 K, the attention level is not positively related to illumination. EEG values under 1000 lx are the strongest at most time, while EEG values in 300 lx are stronger than those in 500 lx in most time. The attention state seems to be the worst under 500 lx; however, it becomes the strongest from the 5th to the 10th minute. During the 10th to the 25th minute, the EEG values decrease and maintain at a low level. Even though it increases starting from the 25th minute, this rise lasts for less than 10 min and then decreases. The attention state keeps steady under 300 lx for the last 30 min. The EEG value under 1000 lx goes through two obvious climbing processes, one is in the 5th to the 20th minute, and the other one is in the 30th to the 40th minute. 

Wilcoxon signed ranks test is used to check whether a significant difference exists in the EEG power value in each 5-min segment, and the average EEG power value of the whole 45 min under paired controlled illumination conditions. According to the detailed data shown in Table 3, the results are all above the significant level at 0.05, which shows that no significant difference is revealed in the EEG values from paired controlled illumination conditions. 

### 3.2. Attention Analysis under Different Color Temperature Conditions

**Figure 4:**
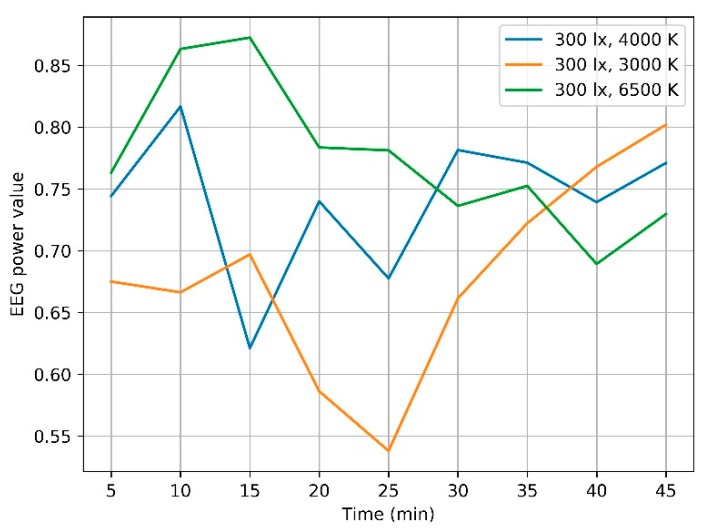
Attention status under different color temperature conditions.

The subject in the environment with the color temperature at 6500 K achieves the strongest EEG value in the first 25 min. In the later 15 min, the color temperature at 4000 K helps to perform better than at 6500 K. However, during these 15 min, the EEG value is the strongest under 4000 K in the former 10 min. The lighting condition at 3000 K only exerts the predominance on the subjects’ attention state for the last 5 min. When focusing on the variation, there is a general decrease when color temperature is set at 6500 K. Compared to this trend, EEG values under 3000 K and 4000 K all exhibit an inverted U shape. When the color temperature is set at 4000 K, EEG values decrease in the first 15 min and increase in the later 30 min. At 3000 K, the reduction lasts for the first 25 min, but the rising is more rapidly, and a higher value has been achieved in the last five minutes. The variations are shown in Figure 4.

Wilcoxon signed ranks test is also used to check the significant difference in EEG power values under paired controlled color temperature conditions. Detailed results shown in Table 4 are all above the significant level at 0.05, which means no significant difference is revealed in either the EEG values in each 5-min-long segments or the average EEG values throughout the learning task under paired controlled illumination conditions.

### 3.3. Post-Interview

**Figure 5:**
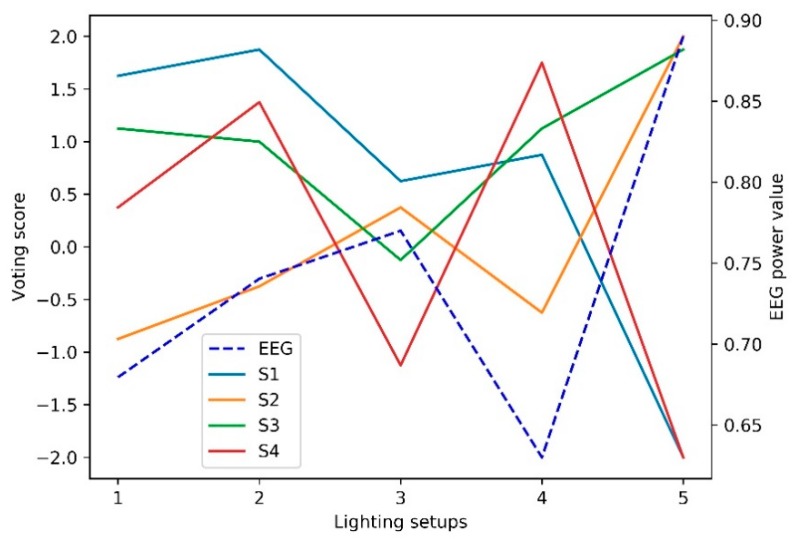
Voting scores and EEG values under different lighting conditions.

The scores of the statements are shown in Figure 5. When illumination is fixed at 300 lx, it gets the highest scores in the aspect of comfort (S1) with the color temperature setting at 4000 K, while the color temperature at 6500 K brings the worst feelings to the subjects. By checking their tendentiousness to keep concentration under the present lighting condition (S3), the scores under 3000 K and 4000 K are similar and significantly higher than those at 6500 K. Considering the satisfaction (S4) throughout the learning task, scores under 4000 K are the highest, then comes the scores under 3000 K. Color temperature at 6500 K drives the worst satisfaction. The perceptions of fatigue (S2) enhance with the color temperature increasing. However, these have no correlation with illumination according to the lowest score at 500 lx and the highest score at 1000 lx. In addition, this perception is positively correlated with attention status (EEG).

All subjects claim uncomfortableness (S1) and dissatisfactory (S4) when the illumination is set at 1000 lx; however, these negative feelings do not restrain their willingness of the concentration for continuous learning tasks (S3). The scores get even higher than those under the other two conditions. Besides, based on the scores under 300 lx and 500 lx, lower illumination wins more scores on comfort but less on satisfactory. The scores are similar in the subjects’ perception of concentration. 

## 4. Discussion

Illumination and color temperature are two popular factors of lighting conditions when digging the human-lighting interaction. This interaction varies in many aspects. People may have different physical and psychological responses to the light environment, and these responses directly lay an effect on human working and learning performance. EEG-based measurement instead of test-score-based measurement provides an objective and practical method to assess the human performance, especially in the occasion where no statistical ‘scores’ can be given.

From the curve shown in Figure 3 and Figure 4, the EEG values vary with the lighting settings. There seems to be the best lighting setup in each segment for subjects’ learning performance. For example, the average EEG value for each five-minute-long segments under the lighting condition with illumination at 1000 lx and color temperature at 4000 K are always the highest during the last 35 min. Under the dim, cold (6500 K, 300 lx) lighting condition, EEG values get higher than those under the other two color temperature conditions in the first 25 min. This seems to indicate that there might be some laws of the effect of lighting environment on human learning performance. However, no significant difference is in the EEG values under two paired controlled lighting conditions, which shows neither the illumination nor the color temperature has a statistical effect on learning performance. This result is in good agreement with Mott’s work in 2012 [7]. Based on the subject-within field study, no effect was shown on human concentration. In his research, the subjects are 84 grade-three children aged 7–8; the lighting conditions were set at 500 lx, 3500 K and 1000 lx, 6500 K. The work highlighted that research with elder subjects need to be done to verify the conclusion. Our work provides strong evidence that attention of people in their 20s has not been affected by lighting conditions ranging from 300 lx to 1000 lx with 4000 K, 3000 K–6500 K with 300 lx. 

Mills et al. [10] conducted a subject-within experiment to investigate the effect of high color temperature on human functioning and working performance. The color temperature is set at 17,000 K and 2900 K. He concluded that high color temperature could improve wellbeing and productivity significantly. The reason of the contradictions was presumed to be that the color temperature ranges of both studies are so narrow that conclusions are partial and limited. 

From Figure 5, subjects’ perceptions of comfort and satisfaction are negatively correlated with EEG values with an exception at dim light with low color temperature condition (300 lx, 3000 K). Subjects’ fatigue is positively correlated with EEG value. Interestingly, subjects strongly affirm the concentration on continuous work despite the intense fatigue, discomfort, and dissatisfaction generated by the high illumination (1000 lx, 4000 K). Smolders et al. [8] conducted a subject-within experiment to study the effect of high illumination on human mental status. The lighting conditions are set at 200 lx and 1000 lx with color temperature at 4000 K. The result demonstrated that alertness, vitality as well as objective performance and physiological arousal could be improved at the 1000 lx condition. This explains why subjects still affirm further concentration with intense negative perceptions. However, the effect induced by high illumination cannot sustain limitlessly. Further work needs to investigate the effective duration for possible utilization in a specific situation.

With the result that neither illumination nor color temperature has a significant effect on computer-based learning efficiency, visual comfort becomes the key factor that contributes to the final conclusion. We suggest that light condition 2 with the set point at 300 lx, 4000 K to be the recommended condition for computer-based learning scene for occupants aged in 20s. Although attention status is the highest at lighting condition 5, the dissatisfaction and discomfort are too high, which is disadvantageous for long-term learning. Lighting condition 3 supports the highest EEG value in the remaining conditions but more satisfaction and comfort are sacrificed for slight improvement compared with light condition 2. Meanwhile, condition 2 is the most energy-saving friendly setting among the conditions recommended by the national standard for lighting design of buildings. This result derived from the experiment with a specific scene and specific setting filled the gap in the whole lighting feature space and human feature space, making a reasonable recommendation to the lighting setting in university architecture with learning function. 

## 5. Conclusions

In this study, EEG-based assessment and interview are conducted to investigate the computer-based learning performance under the 5 lighting conditions. Main conclusions from this study include:

1. The attention of people aged in the 20s is not affected by the investigated lighting conditions;
2. People in the high illumination at 1000 lx are more capable of sustaining attention despite the discomfort and dissatisfaction;
3. Taking the EEG results and post-interview answers into consideration, the lighting conditions at 300 lx, 4000 K is the recommended setting in university architecture with learning function.

However, this research has the following limitations. Firstly, while choosing the subjects, the gender ratio hasn’t been taken into consideration. Further study should get female subjects involved to check whether the effect is gender-related. Secondly, building lighting contains many aspects, more than illumination and color temperature. The lighting device types and locations matter, as well. These aspects should be integrated into the experiment design for a specific scene.

## Figures and Tables

**Table 1:** Lighting setups in the experiments.

No.	Lighting Setup	Set Points	Actual Value
1	dim, warm	300 lx, 3000 K	307 lx, 3122 K
2	dim, white	300 lx, 4000 K	308 lx, 4180 K
3	dim, cold	300 lx, 6500 K	308 lx, 6415 K
4	neutral, white	500 lx, 4000 K	502 lx, 4180 K
5	light, white	1000 lx, 4000 K	1005 lx, 4180 K

**Table 2:** Statements in the interview.

No.	Statements
1	I feel comfortable about the present lighting condition
2	I feel exhausted under the present lighting condition
3	I can concentrate for a continuous work for another 15 min
4	I am satisfied with the present lighting condition during learning

**Table 3:** Significance difference test results under various illumination conditions.

Segments	300 lx–500 lx	500 lx–1000 lx
1st	0.401	0.09
2nd	0.779	0.499
3rd	0.123	0.398
4th	0.575	0.237
5th	0.327	0.612
6th	0.779	0.237
7th	0.779	0.091
8th	0.484	0.499
9th	0.889	0.237
45-min average EEG	0.889	0.31

**Table 4:** Significance difference test results under different color temperature conditions.

Segments	3000 K–4000 K	4000 K–6500 K
1st	0.889	0.575
2nd	0.263	0.674
3rd	0.674	0.263
4th	0.263	0.484
5th	0.401	0.575
6th	0.575	0.401
7th	0.889	0.889
8th	0.674	0.889
9th	0.866	0.889
45-min average EEG	0.674	0.575
